# Repurposing Inflatable Packaging Pillows as Bioreactors: a Convenient Synthesis of Glucosone by Whole-Cell Catalysis Under Oxygen

**DOI:** 10.1007/s12010-020-03448-x

**Published:** 2020-11-13

**Authors:** Michael D. Mozuch, Kolby C. Hirth, Thomas J. Schwartz, Philip J. Kersten

**Affiliations:** 1 Forest Products Laboratory, Forest Service, US Department of Agriculture, Madison, WI 53726 USA; 2 Department of Chemical and Biomedical Engineering, University of Maine, Orono, ME 04469 USA

**Keywords:** Whole-cell catalysis, Bioreactor, Oxygen limitation, Biorefinery, *Phanerochaete chrysosporium*, Pyranose 2-oxidase, Catalase, Glucosone

## Abstract

Biocatalysis using molecular oxygen as the electron acceptor has significant potential for selective oxidations at low cost. However, oxygen is poorly soluble in water, and its slow rate of mass transfer in the aqueous phase is a major obstacle, even for laboratory-scale syntheses. Oxygen transfer can be accelerated by vigorous mechanical methods, but these are often incompatible with biological catalysts. Gentler conditions can be achieved with shallow, high surface area bag reactors that are designed for single use and generally for specialized cell culture applications. As a less-expensive alternative to these high-end bioreactors, we describe repurposing inflatable shipping pillows with resealable valves to provide high surface area mixing under oxygen for preparative synthesis of glucosone (D-arabino-hexos-2-ulose) from D-glucose using non-growing *Escherichia coli* whole cells containing recombinant pyranose 2-oxidase (POX) as catalyst. Parallel reactions permitted systematic study of the effects of headspace composition (i.e., air vs 100% oxygen), cell density, exogenous catalase, and reaction volume in the oxidation of 10% glucose. Importantly, only a single charge of 100% oxygen is required for stoichiometric conversion on a multi-gram scale in 18 h with resting cells, and the conversion was successfully repeated with recycled cells.

**Supplementary Information:**

The online version contains supplementary material available at 10.1007/s12010-020-03448-x.

## Introduction

Molecular oxygen is considered the “greenest” oxidant in organic synthesis, and it has garnered increasing interest both in large industrial-scale syntheses and in fine chemical manufacture [1–3]. Oxygen is abundant and inexpensive, and the product of its reduction is ultimately water, thereby decreasing environmental impacts and increasing atom efficiency in synthesis. One challenge with the use of oxygen in chemical synthesis is its limited solubility in solvents, particularly water, which leads to reaction rates that are controlled by the rate of mass transfer of oxygen from gas to liquid phase rather than on intrinsic kinetics [4]. Chemical catalysis is also limited in its ability to achieve regio- and stereo-specificity, which often precludes its use in the manufacture of biologicals. Oxygen-dependent enzymes provide an alternative to chemical catalysts and have potential advantages in achieving selective oxidations in an environmentally sound manner [5, 6]. However, these reactions suffer the same reaction rate limitations associated with oxygen transfer from gas to liquid phase. Aggressive mechanical dispersive methods to increase oxygen transfer often inactivate enzymes and whole-cell catalysts, thereby making reactor design to address these issues an active area of research [7, 8].

**Fig. 1:**
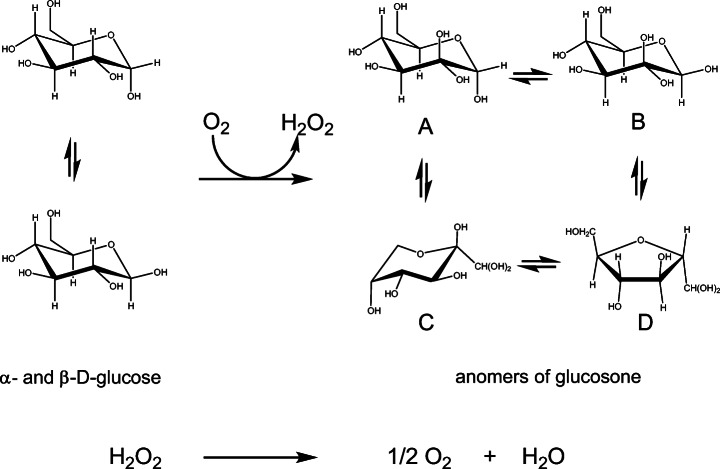
Reactions catalyzed by POX and catalase. Both α- and β-D-glucose are oxidized at C-2 by POX to produce glucosone and peroxide (upper equation). The glucosone equilibrates as four isomers in water. The peroxide is deleterious to the POX reaction and can be consumed using catalase (lower equation)

To address this challenge and demonstrate the utility of an inexpensive bag reactor, we focus on the synthesis of glucosone from glucose on a multi-gram scale with molecular oxygen as the two-electron acceptor for oxidation of glucose, and with pyranose 2-oxidase as the regio-selective catalyst (Fig. 1). Pyranose 2-oxidases are studied for the oxidation of not only glucose but also of related sugars for the synthesis of rare keto sugars and fine chemical synthons [9–14]. Glucosone has been applied in the enzymatic [15] and chemo-enzymatic [16, 17] synthesis of fructose and in the chemo-enzymatic synthesis of furylglycolic acid [18]. Fungal pyranose 2-oxidases (POX; EC 1.1.3.10) are members of the functionally diverse glucose-methanol-choline (GMC) superfamily [19] and are proposed to play a role in lignocellulose metabolism by supplying peroxide for peroxide-dependent enzymes [20–22]. These oxidases comprise, along with other fungal GMC enzymes, the auxiliary activities family AA3 of the CAZy database of carbohydrate-active enzymes, with POX in (sub)family AA3_4 [23]. Pyranose 2-oxidases are found in diverse basidiomycetous fungi [19, 24], and a subset of these fungi also express pyranosone dehydratase, which converts glucosone to cortalcerone that is proposed to function as an antibacterial agent suppressing competition for wood decay fungi [25, 26]. In addition to decay wood basidiomycetes, POX has also been characterized from ascomycetes [27] and bacteria [28, 29], and therefore, there is a broader context of possible catalysts that might be used for sugar oxidations with molecular oxygen. For the present study, we use *E. coli* whole-cell catalysis with recombinant POX from the white-rot wood decay fungus *Phanerochaete chrysosporium*. The corresponding *pox* cDNA from this basidiomycete codes for 621 amino acids [21], and the recombinant protein includes an N-terminal T7-epitope and C-terminal hexahistidine tag [30]. The 270-kDa oxidase is homotetrameric and consists of four identical 68-kDa subunits, each with a flavin adenine dinucleotide active site [31].

Whole-cell catalysis can have significant advantages over catalysis with purified enzymes [32–34]; however, it can be challenging to accomplish selective glucose upgrading by this approach because of the competitive consumption of glucose by constitutive metabolism of active cells. One potential solution to this issue is the application of “resting” cells, an intermediary option in a continuum of possibilities that range from cell-free processes to whole-cell fermentations [35]. “Resting” or “non-growing” cells describe those that have been isolated (typically by centrifugation) and re-suspended in a defined solution that is deficient in requirements for growth. There are many examples of the application of resting cells for conversion of diverse substrates in single-step syntheses [36–38]; however, the use of glucose as a “sacrificial” substrate (e.g., to regenerate a cofactor) provides the most insight on how resting cells can be effective in glucose transformation [39–41]. For example, resting cells in M9 medium minus a nutrient nitrogen source can use central metabolism to regenerate NADPH for Baeyer–Villiger oxidation of cyclohexanone in the presence of supra-stoichiometric glucose [39]. By contrast, oxidation of glucose can be tightly coupled to the reduction of cinnamyl aldehyde in resting cells with recombinant glucose dehydrogenase and an alcohol dehydrogenase when reactions are carried out in phosphate buffer [41].

We previously reported whole-cell catalysis with POX in phosphate buffer using stoppered Erlenmeyer flasks for the chemo-enzymatic synthesis of furylglycolic acid [18]. A headspace consisting of pure oxygen is important for these reactions to compensate for the poor solubility of O_2_ in water. However, if the reaction is carried out in a fixed-volume batch reactor with only an initial charge of O_2_, the combination of high glucose concentrations coupled with small headspace volumes leads to a significant drop in partial pressure of oxygen as the reaction progresses, ultimately decreasing the rate of mass transfer and, correspondingly, of oxidation. This limitation can be ameliorated by repeatedly charging the headspace with oxygen, or by using a large headspace volume relative to the reaction volume to minimize the pressure drop, but the inconvenience of these approaches is compounded with long reaction times and parallel reactions. Continuous headspace exchange, such as with sparged stirred-tank reactors, is an alternative that can be even more technically challenging when performing multiple simultaneous reaction studies (e.g., with 12 simultaneous reactions as used in our studies here with pillow reactors). Because of the very low solubility of O_2_ in water (e.g., 0.0013 mol L^−1^ for 1 bar of O_2_ at 298 K), such approaches do not completely eliminate mass transfer limitations, and measured reaction rates (or selectivities) thus depend on aeration and agitation conditions that must be faithfully replicated for all reactions. This study investigates the use of variable volume reactors—inflatable/collapsible shipping pillows—which may provide a convenient alternative to rigid-walled reactors, similar to the application of bag reactors in mammalian or insect cell culture [42], but at significantly lower cost and with a simpler configuration because headspace gas exchange is not required. For the purposes of our discussion, we will use “bag” and “pillow” (bio)reactors interchangeably.

**Fig. 2:**
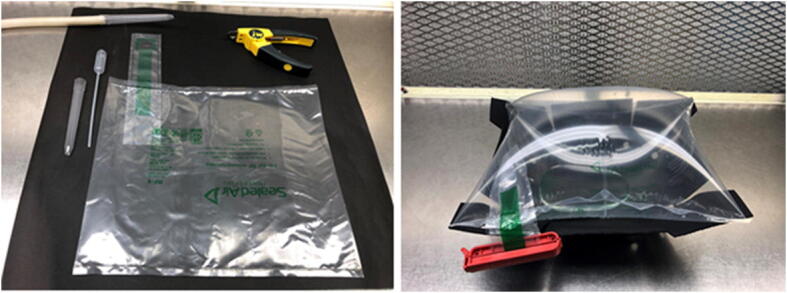
Shipping pillow adapted as a bioreactor. The uninflated Fill-Air^TM^ RF-1 shipping pillow and accessories are shown in the left panel. A 5-ml pipet tip with the narrow end clipped (using clippers shown upper right) is used as a funnel once it is fully inserted through the resealable valve. After addition of the liquid reaction suspension, the pillow is inflated with air or oxygen using a fresh pipet tip fitted to the end of C-Flex® tubing (upper left). For single-time point determinations, a corner of the bioreactor is cut to remove the contents. For time course studies, samples are removed through the valve with a transfer pipet, shown immediately left of the shipping pillow. The right panel shows an inflated pillow containing 50 ml of water, for demonstration, in a support made of Kydex® V thermoplastic (see “Methods”). The red clip on the external valve extension is used as a precaution against leaks

Inspired by the work of Bwambok et al. [43] describing the use of bubble wrap for analytical methods and to grow microorganisms, we tested inflatable shipping pillows as bioreactors that might circumvent issues related to the use of rigid-walled bioreactors. The Fill-Air^TM^ RF-1 shipping pillow appeared to be a good candidate for our application because it has a resealable valve, which is designed for manual inflation with pressurized air, and this valve can be used to introduce both a liquid reaction suspension and a defined headspace gas (Fig. 2). The desired properties for our bioreactor were (1) high oxygen concentration in the headspace for extended reactions at high glucose concentrations using a single initial charge of oxygen, (2) high air-liquid interfacial area to facilitate oxygen transfer to the aqueous phase, (3) gentle mixing with no sparging to minimize cell and enzyme inactivation, and (4) simplicity of operation that would allow multiple experiments to be run in parallel at minimal cost in both expendables and equipment. Procedures to repurpose the shipping pillow as a bioreactor are described here for the oxygen-dependent conversion of glucose to glucosone catalyzed by POX from *P. chrysosporium* expressed in *Escherichia coli* (Fig. 1). When applied to resting cell catalysis under non-growth conditions, the method provides a convenient, inexpensive, and effective alternative to commercial high-end inflatable and fixed-volume bioreactors. Broad application of the methods described here may be suitable for use with other oxidizing enzymes [44] and whole-cell catalysts [45].

## Materials and Methods

### Materials

Catalase purified from bovine liver and D-arabino-1,4-lactone were purchased from Sigma-Aldrich. Fill-Air^TM^ RF-1 inflatable packaging pillows from Sealed Air® were purchased from Uline (model S-5234; 9 × 11 in., 0.2 cu. ft). Maxi Pipet Tips (#21-195-3; 1000-5000ul), transfer pipets (#13-711-6M), and molded fiberglass trays (MFG Tray, Linesville, PA; model #920108; 29.84 × 22.22 × 10.41 cm; Fisher Scientific Cat. No. 15-239-33) were purchased from Fisher Scientific. C-Flex® tubing (# SK-06424-74; 1/4"ID x 1/2"OD) was purchased from Cole-Parmer®. Kydex® V thermoplastic (0.060″ thick–12″ × 12″; manufactured by Sekisui SPI) was purchased through Amazon.com. Plaster cloth gauze was purchased from a local hobby shop.

### Preparation of Bioreactor Supports

A fully inflated Fill-Air^TM^ RF-1 pillow requires a support so as to allow the pillow to move in coordination with the incubator shaker, as opposed to rocking in place. Supports were made by first making a cast of an inflated pillow with plaster gauze (the cast is made to half of the inflated pillow), and then the cast was used to make pillow supports from Kydex® V thermoplastic. Eight tapered notches, about 3 in. long and 1 in. wide at the edge of the sheet, were cut into the 12 × 12 in. thermoplastic sheets in a radial pattern to help the thermoplastic sheet conform to the shape of the cast without buckling (Additional file 1: Figure S1). The notched thermoplastic sheets were then preheated at approximately 170 °C until pliable before pressing into the plaster cast supported in a bed of sand. The formed sheet was then trimmed with a shears and the shape fine-tuned using a heat gun so that it would fit in the MFG tray (Additional file 1: Figure S1).

### Bacterial Strain and Plasmids

The cDNA sequence (GenBank accession number AY522922) encoding POX of *P. chrysosporium* BKMF-1767 [21], with an N-terminal T7-tag and a C-terminal His6 tag [30], was synthesized for optimized expression in *E. coli* and inserted in pJ414 (ATUM, Newark, CA, USA). A control pJ414 plasmid with no open reading frame in the insert was also prepared, and both plasmids were transformed into BL21(DE3) *E. coli*.

### Growth and Preparation of Cells for Catalysis

Cultures were grown overnight on non-inducing medium to inoculate auto-induction medium based on that of Studier [46] with modifications [47]. In brief, each liter of auto-induction medium contained 48 g terrific broth (TB) powder, 2 mM MgSO_4_, 0.2X trace metals (1000X trace metals, Teknova Cat. No: T1001), 28 mM succinic acid, 0.005% antifoam 204, 8 g glycerol, 0.15 g glucose, and 5 g lactose. The glycerol, glucose and lactose were autoclaved separately before addition to the medium. Filter-sterilized carbenicillin (100 μg ml^−1^ final) provided selection. Five hundred milliliters of auto-induction medium in 2-l baffled flasks was inoculated with 3 ml of non-induced culture and incubated at 250 rpm, 20 °C for 30 h. Cells were harvested as 40-ml aliquots in 50-ml conical centrifuge tubes at 4000×*g* for 15 min. The supernatants were removed, and the cell pellets overlaid with 10 mM citrate pH 6 buffer before storage at − 20 °C.

### Reaction Conditions

All reactions in this study were at 10% glucose in 10 mM pH6 sodium citrate. Catalase was added at levels indicated. A fiberglass tray was used to contain the repurposed Fill-Air^TM^ RF-1 bioreactors with or without a Kydex® V thermoplastic support (see “Results and Discussion”). Incubations were at 100 rpm and 22 °C using standard laboratory incubators with a 1″ rotational diameter. A corner of the bioreactor was cut to release the contents, or a transfer pipet used to remove samples for time course experiments (Fig. 2).

To prepare the BL21(DE3) *E. coli* cells for catalysis, 50-ml centrifuge tubes with frozen pellets and overlays were centrifuged to maintain the cell pellets as the overlays thawed (about 15 min) and the supernatants discarded. The cell pellets were then washed twice by suspending each pellet in 40 ml of 10 mM pH6 sodium citrate and centrifuging at 4000×*g* for 15 min and the supernatants discarded. The washed pellets were then suspended in reaction solutions at cell densities dictated by the purpose of the experiment. For easy comparison of cell densities used in various experiments, we present cell densities as a fraction compared to the cell density of culture at the time of harvest (55 g wet weight cell l^−1^ culture).

### Enzyme Assays

POX activity in the cell extract was determined in a peroxidase-coupled assay as described elsewhere [30], where one unit of POX is defined as the amount of enzyme required to oxidize 1 μmol of glucose per minute.

To prepare *E. coli* cell extract for the determination of POX activity, a cell pellet (approximately 2.2 g wet weight) from 40 ml of culture was sonicated (Qsonica Model Q700) in 10 ml of 50 mM Tris pH 8, 50 mM NaCl, and 5% glycerol for a total processing time of 20 min and the sonicate centrifuged. Based on the assay of the cell extract, the POX activity was estimated to be 4500 U l^−1^ culture, comparable to the previous report of POX activity in cultures of BL21(DE3) *E. coli* transformed with *pox* in pET21a(+) [30].

Catalase activity was determined at 240 nm in 50 mM Tris-HCl buffer (pH 9.0) using 10 mM H_2_O_2_ and an extinction coefficient for H_2_O_2_ of 39.4 M^−1^ cm^−1^ [48]. One unit of catalase is defined as the amount of enzyme required to decompose 1 μmol of peroxide per minute. The specific activity of the catalase in our studies was 3800 U per mg protein.

### NMR Spectroscopy

NMR experiments were run on a Bruker Avance III HD 500 MHz spectrometer fitted with a 5-mm Prodigy triple resonance inverse-detection TCI cryoprobe with z-gradients. The quantitative ^13^C-NMR experiments used the standard Bruker pulse program with an inverse-gated proton-decoupling sequence, a 30^o^ pulse angle (4 μs) and a 90 s relaxation delay. Spectra were recorded with 64 k data points and a 236 ppm spectral window. Exponential multiplication (LB = 1.0) and one level of zero-filling were performed prior to Fourier transformation. All samples were run in H_2_O:D_2_O mixture, and spectra were referenced using the Bruker default solvent table with no reference shift for D_2_O. Peaks were picked using the automatic routine and a minimum intensity of 0 and a peak detection sensitivity of 1.40.

### NMR Data Analysis

Peak tables were exported to Excel for comparison of peak heights. For each component in a particular sample, peak heights of the non-overlapping carbon signals were totaled and then divided by that number of carbons so as to obtain the relative ratio of components. The relative ratios were scaled so that their total was 100 and then graphed as a percentage of total. The chemical shift assignments for glucosone were based on reported values [49].

### HPLC Analysis

An Agilent 1200 series HPLC instrument equipped with a Phenomenex Rezex (Torrance, CA) RCM-Monosaccharide Ca^2+^ column at 85 °C, a water mobile phase (0.3 ml/min), and a refractive index detector (RID) was used to quantify glucose and glucosone. An equivalent response factor for glucose and glucosone was used for quantitation [50].

## Results and Discussion

### Repurposing Shipping Bags with Resealable Valves

Although repurposing a shipping pillow as a bioreactor is simple in concept, there are procedural details worth description. For the original intended use of the Fill-Air^TM^ RF-1 shipping pillows, an inflator nozzle is used to inflate the pillow under pressure through the extended pillow valve (Fig. 2). Upon removal of the nozzle, the valve reseals when gentle pressure is applied. However, as a bioreactor, a reaction solution is first introduced into the pillow before the headspace gas. This can be achieved with the aid of a disposable 5-ml Maxi Pipet Tip that is cut at the narrow end for easy flow of liquid under gravity. The pipet tip serves two functions, as a funnel to pour the liquid reaction mixture and as a spacer that separates the valve tube that extends into the bag so the liquid flows freely. Therefore, it is important that the pipet tip extends into the bag until it fits snuggly into the ¾ in. valve opening as liquid is poured into the pillow. Upon removal of the pipet tip, the valve opening is gently pinched to reseal the valve. At this point, the reaction medium is contained in the pillow with essentially no headspace because the liquid is introduced into a deflated pillow (supplied from the manufacturer without any gas in the pillow). Gas (air or oxygen) under pressure is then introduced into the pillow using a fresh Maxi Pipet Tip as a nozzle (as before, with the narrow end cut). The C-Flex® tubing used as an inflation hose has a ½ in. outside diameter that fits snuggly inside the wide end of the pipet tip, allowing easy exchange of tips as needed (Fig. 2). After inflation of the bioreactor, the tip is removed and again the valve pinched to reseal the pillow. As a precaution, a clip (as is commonly used for dialysis bags) is used as a secondary seal at the external valve extension (Fig. 2).

For our initial studies, an incubator shaker was used to rotate the bioreactor causing the aqueous phase to travel up the sides of the pillow (Additional file 2: Movie S1). A support for the pillow was made of Kydex® V thermoplastic, and when placed in a close-fitting container, the support prevents the reactor from independently rocking and also helps maintain the parabolic surface for the reaction solution (Additional file 1: Figure S1). However, even with the Kydex® support, the mixing patterns of the system change as the reaction progresses and the bag deflates. Efforts to maintain more consistent mixing patterns (e.g., with an applied pressure to the bag) are not described here for the purposes of having a simple and effective method for complete reaction at 18 h. A frequency of 100 rpm (1″ rotational diameter) for the incubator was chosen based on visual inspection of the extent of mixing, with the aim being to maximize travel of the 50-ml solution up the sides of the reactor at initial reaction conditions.

### Preliminary Studies

As guidance for a suitable glucose concentration and incubation time for reactions, we looked to previous work where complete conversion of glucose was achieved at a gram-scale or higher. Early pioneer work with immobilized pyranose 2-oxidase from *Polyporus obtusus* demonstrated complete conversion of 2 g/l glucose in 24 h under air [51]. More recent work reports complete conversion of 1 g/20 ml (i.e., 5% w/v) glucose with pyranose oxidase from *Trametes hirsute* in 24 h under air [15]. Leitner et al. observed complete reaction for 200 ml of 250 mM glucose in only 5 h with POX from *Trametes multicolor* when pure oxygen was supplied through a sintered glass tube [16]. The report of the highest glucose concentration we could find in the literature, as well as the longest incubation time, was in a roller mixer with 5 g of glucose dissolved in 20 ml water, incubated for 48 h with aeration of the solution using compressed air every hour for 3 min [52]. Based on these reports, and the desire that the reactions should be unattended, we chose 18-h (overnight) incubation times and an initial glucose concentration of 10% glucose (i.e., 555 mM). Also, in contrast to the earlier studies with purified pyranose 2-oxidases, we used whole-cell catalysis with *E. coli* producing intracellular pyranose 2-oxidase from *P. chrysosporium*.

For our studies here, cells from frozen pellets (see “Methods”) were washed twice with 10 mM citrate prior to reaction with glucose for the following reasons: (1) to remove components of the rich growth medium, (2) washing may improve permeability particularly when a rich growth medium is used [53], and (3) to remove citrate complexed metals that may contribute to Fenton-type reactions in the presence of peroxide generated by POX [54].

Our preliminary experiments were at the 50-ml reaction scale using cells harvested from 40 ml of culture, or a cell density of 0.8 in reference to cultures at time of harvest. The cells corresponding to 40 ml culture had a wet weight of approximately 2.2 g and activity of 190 U as determined by assaying supernatants of sonicated cells (see “Methods”). In our earlier syntheses of glucosone by whole-cell catalysis in Erlenmeyer flasks, we used catalase at 50 μg ml^−1^ reaction solution [18]; therefore, we tested catalase levels at 50 μg ml^−1^ and also at 100 μg ml^−1^ to determine if sufficient catalase was being used to protect the whole-cell catalysts in 18-h incubations with a headspace of 100% oxygen. Reactions in triplicate at both catalase levels were complete as determined by quantitative NMR, which requires assignment of all the anomeric species (Fig. 1).

These preliminary experiments indicated that transport of glucose into the *E. coli* cells (prepared from frozen cell pellets) was accomplished. By contrast, fresh cells import extracellular glucose into the periplasmic space across the outer membrane porins (OmpC, OmpF, and OmpB), but the internal membrane is impermeable to glucose, and active transport systems are used to transport sugar to the cytoplasm (reviewed: [55]). The phosphoenolpyruvate (PEP) phosphotransferase system (PTS) is the dominant transporter of glucose with concomitant conversion of glucose to glucose-6-phosphate which feeds into the central metabolism [33, 56, 57]. Therefore, glucose import is intertwined with active intracellular glucose metabolism because of the PEP requirement for PTS. However, cells can be made permeable to glucose with a freeze-thaw cycle. For example, “fresh” *E. coli* BL21 cells with a glucose dehydrogenase (GDH) from *Bacillus subtilis* had no GDH activity, but frozen and thawed cells exhibited activity, indicating that there was partial permeabilization of cells due to the freeze-thaw process [40].

It is generally recognized that freeze-thaw cycles make cells leaky and can improve whole-cell catalysis [58] and that cell membrane damage is dependent not only on the rates of freezing and thawing but also on the nature of the solutes in the suspending medium [59–61]. Washing cells in buffer also increases permeability and enhances catalysis with resting cells [53, 62], but to our knowledge, whether this applies to glucose permeability has not been systematically studied. A systematic investigation of all factors that may contribute to membrane permeability was beyond the scope of the present study. Based on the success of our preliminary studies, we proceeded using cells that underwent a single freeze-thaw cycle, were washed twice in a citrate buffer just prior to catalysis, and provided a constant catalase concentration of 50 μg ml^−1^ reaction.

### Reaction as a Function of Cell Density and Headspace Composition (Oxygen vs Air)

**Fig. 3:**
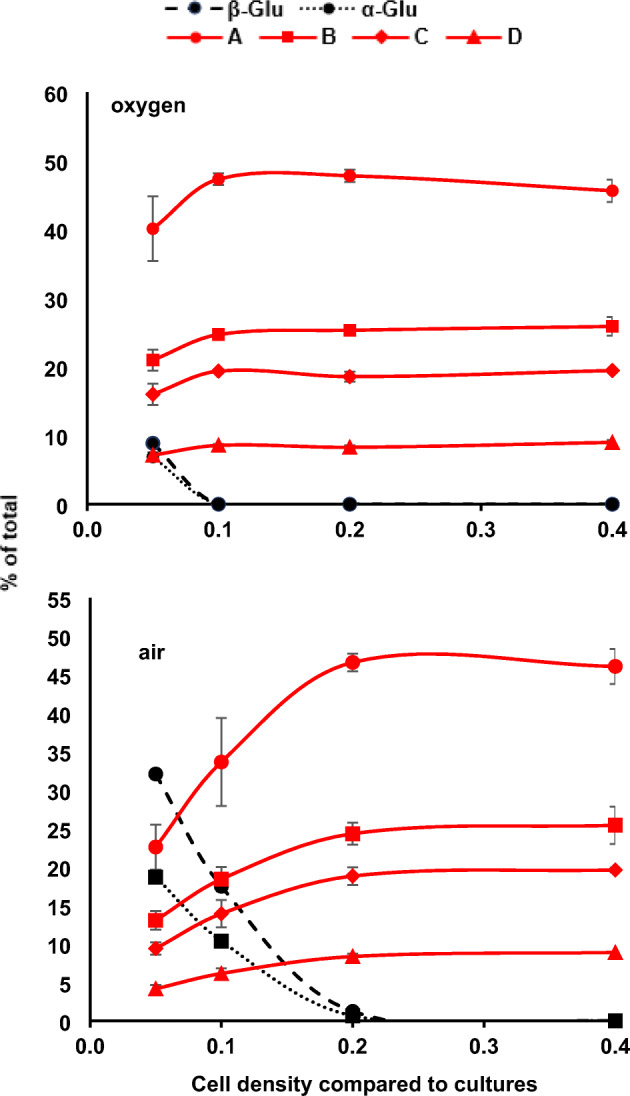
Glucose consumption and glucosone production as a function of POX cell density. Reactions were run in triplicate at cell densities indicated under oxygen (upper graph) or air (lower graph) headspace. The α-D-glucose (α-Glu), β-D-glucose (β-Glu), and glucosone anomers (see Fig. 1 for anomer letter designations) were quantified by NMR. Cell densities are with respect to that of culture at time of harvest (55 g wet weight cells l^−1^ culture). The catalase concentration was 50 μg ml^−1^

A series of pillow reactions with 50 ml of 10% glucose were run in triplicate with twofold serial dilutions of cells, the catalase concentration at 50 μg ml^−1^, and with the headspace filled with either 100% oxygen or air. Reactions were harvested at 18 h and samples analyzed by quantitative NMR (Fig. 3). Surprisingly, reactions were complete in 18 h even with only 0.1 cell density (compared to growth culture cell density) under oxygen and with 0.2 cell density under air.

Since the bioreactor in this simple arrangement is a closed system and has a single charge of headspace at the beginning of the reaction, the stoichiometry of the reaction requires consideration (Fig. 1). For example, in the 50-ml reaction volumes with 10% glucose, there are 0.028 moles of glucose requiring 0.028 moles of oxygen. However, the dismutation of the peroxide by catalase generates oxygen. The 0.028 moles of oxygen consumed generates 0.028 moles of peroxide which dismutates to provide 0.014 moles of oxygen per the lower equation of Fig. 1. Accordingly, a minimum of 0.014 moles of oxygen must be provided in the headspace to achieve oxidation of 5 g of glucose to glucosone in a coupled reaction with catalase.

The pillow has a nominal volume of 5.6 l (0.2 ft^3^), but the measured displacement volume of a fully inflated pillow is only about 3.2 l. Therefore, the headspace in a pillow inflated with oxygen contains approximately 0.13 moles (assuming an ideal gas at 1 atm), an approximately tenfold excess compared to the minimum required by stoichiometry. Because the pillow is not a rigid vessel, the total pressure in the system remains at approximately 1 atm throughout the reaction. Assuming that the gas exchange across the low density polyethylene bioreactor surface is negligible over the 18-h reaction time scale (which is reasonable, given that typical diffusivities of O_2_ across LDPE membranes are on the order of 1 × 10^−7^ cm^2^ s^−1^ [63]), the bioreactors with 100% oxygen should maintain a headspace of 100% oxygen at approximately 1 atm throughout the reaction. Conversely, if the pillows were inflated with air (21% oxygen), then the pillow bioreactor will hold 0.027 moles oxygen, which is still a twofold excess of that required for complete conversion of 5 g of glucose. However, despite the constant total pressure ensured by the pillow vessel, the oxygen composition of an air headspace will decline from 21 to about 10% oxygen during the course of the reaction, which leads to a decrease in the partial pressure of oxygen and thus a decrease in the oxidation rate as the reactions progress (Fig. 3, lower panel).

### Reactions as a Function of Catalase

**Fig. 4:**
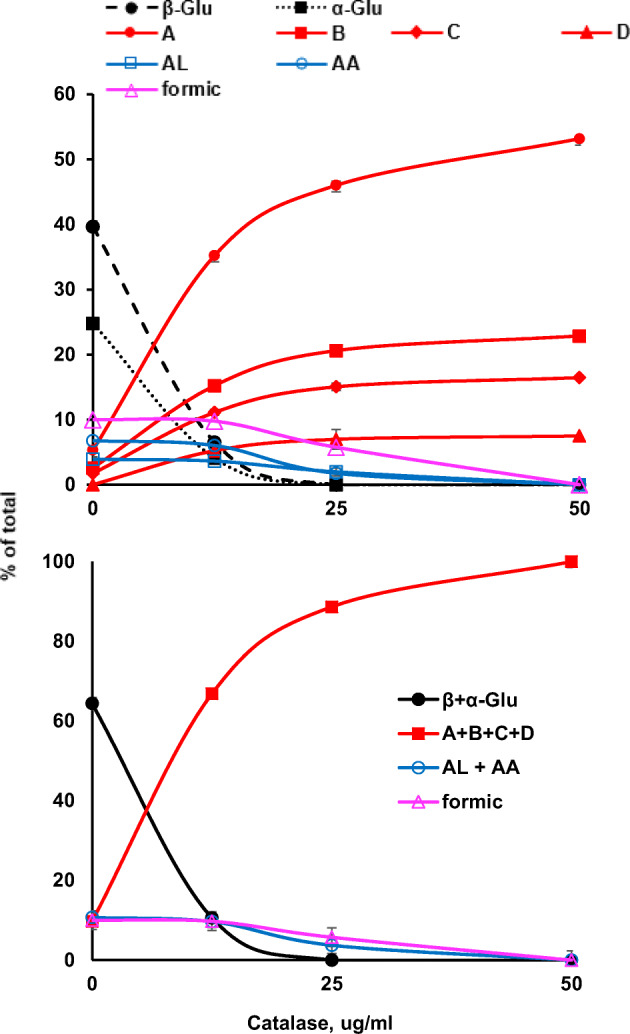
Glucose consumption and product formation as a function of catalase. The upper graph shows the anomeric complexity of glucose and glucosone and the side products of formic acid, D-arabino-1,4-lactone (AL), and D-arabinonic acid (AA) in 18-h reactions. The lower graph plots summations for glucose anomers, glucosone anomers, formic acid, and (AL + AA). Results indicate that near equimolar amounts of formic acid and (AL + AA) are formed at low catalase concentrations, supporting evidence for their formation according to the equation of Fig. 5. With no addition of catalase, there was approximately 5% unidentified product(s) that was not accounted for in these graphs

**Fig. 5:**
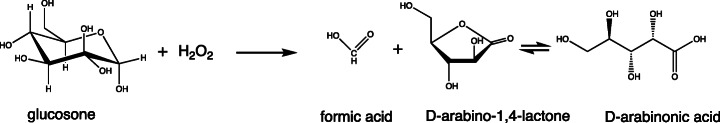
Proposed mechanism for side product formation without exogenous catalase. The production of formic acid, D-arabino-1,4-lactone, and arabinonic acid can be explained by the reaction of glucosone with hydrogen peroxide, as proposed by Vuorinen [64]

We proceeded to test the role of catalase in these reactions using a cell density ratio of 0.2 (compared to cultures) because this provided sufficient cells (about 2 times that required for an 18-h reaction) for complete conversion of glucose under oxygen (Fig. 3). This cell density is equivalent to 0.55 g wet cells per 50-ml reaction, which is 4 times less than in our initial experiments (0.8 cell density compared to culture; see “Preliminary Studies”). Using a twofold series of dilutions of catalase, a strong dependence on catalase for completion of reactions was demonstrated (Fig. 4). Importantly, reactions with no exogenous catalase showed significant levels of D-arabino-1,4-lactone, D-arabinonic acid, and formic acid (Fig. 5). Control reactions with 10% glucose and whole cells prepared in the same manner, except with pJ414 plasmid with no open reading frame, showed no conversion of glucose in the presence of catalase at 50 μg ml^−1^ reaction solution, formally demonstrating that pyranose 2-oxidase is required for conversion of glucose to glucosone and that catalase has a strong influence on the extent of the reaction, presumably by preventing inactivation of pyranose 2-oxidase.

The reaction of glucosone with peroxide to form D-arabino-1,4-lactone, D-arabinonic acid, and formic acid (Fig. 5) is not without precedent in the literature. Vuorinen [64] has shown that cleavage of glucosone is first-order with respect to hydrogen peroxide and hydroxyl ion at low alkalinities. Under the mildly acidic starting conditions (pH 6) of our reactions, this non-enzymatic reaction may have occurred subsequent to significant accumulation of peroxide, thus delaying the drastic drop in pH associated with formic acid and D-arabinonic acid production that would inactivate enzymatic catalysis. Detailed kinetic studies would help to disentangle the possible reaction pathways, including time-dependent changes in post-harvest samples; however, such studies were outside the scope of the present study. The production of D-arabinonic acid and formic acid likely has inhibitory effects on POX activity, and possibly cell integrity, when insufficient catalase is present. A possible alternative to the addition of exogenous catalase would be to use cells that co-express POX and catalase. This strategy of co-expression has been recently demonstrated for production of α-ketoglutarate by whole-cell catalysis with glutamate oxidase and catalase [65].

### Increased Reaction Scale Under Oxygen and Air

Based on the previous analyses on available oxygen in the pillow reactors and the stoichiometry of the reactions, it seemed plausible that reactions under pure oxygen could be scaled successfully to 200 ml in the same bag, but whether the reactions would be complete in 18 h was in question. Reactions run in triplicate for 18 h under oxygen and air showed that 200-ml reactions under oxygen were complete, whereas the reactions under air were a little less than half complete, consistent with oxygen being the limiting reactant under air. Encouraged that reactions could be scaled to 200 ml under oxygen, but that the quality of mixing quickly degraded as the bags deflated, even with use of the thermoplastic supports (Fig. 2), we tested 200-ml reactions under oxygen but without supports. As would be expected, the bags without form-fitted support rolled around the rectangular molded fiberglass containers in the rotary incubator. However, as the oxygen is consumed, the bags flatten and the reactors stabilize, and the mixing becomes much more consistent. As a further test of the utility of the process, cells were recovered by centrifugation and reused in fresh solutions of 10% glucose and catalase. Complete conversion of glucose was observed for an 18-h reaction (Additional file 1: Figure S2). The determination of how many cycles the cells can be reused was beyond the scope of this study, but factors such as the efficiency of cell recovery from the high surface area bioreactors and susceptibility of cell walls to freeze-thaw cycles would likely be important.

**Fig. 6:**
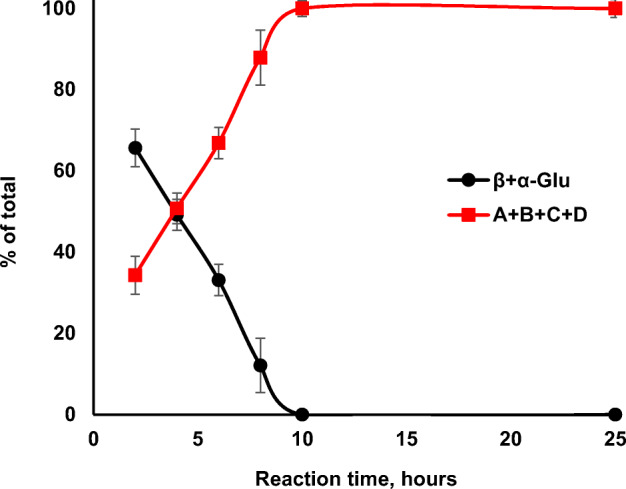
Time course reaction at 200-ml scale under oxygen. The concentrations of glucose and glucosone were followed by NMR in samples removed from 200-ml reactions through the resealable valve with a transfer pipet. Reactions were run in triplicate. The extrapolation to zero time does not fit the theoretical prediction in the y-axis most likely because of the imprecise determination of time zero for the six samples during preparation.

A time course study of 200-ml reactions with no form-fitted support showed the reactions to be complete in 10 h (Fig. 6). Importantly, samples could be removed through the resealable valve without significant deflation of the pillows, and no oxygen was replenished to the pillows for the entire time course and yet the reactions proceeded to completion. Based on these conditions (0.2 l reaction volume, 10 % glucose, completion in 10 h), a volumetric productivity of 9 g (l h)^−1^ is observed, which is remarkably similar to that observed with purified POX from *T. multicolor* in a continuously stirred reactor (200 rev min^−1^) with sparging oxygen through a sintered glass tube [16]. Likewise, the reactions here (Fig. 6) used approximately 1 U POX ml^−1^ reaction (based on estimation of sonicated extract) and provides a specific productivity of 9 mg (l h U POX)^−1^ which is also similar to that observed with the reactor using purified POX [16]. Detailed comparisons have limitations in that these are not kinetic studies, and in one case, whole cells are used while in the other a homogeneous catalyst (i.e., POX). In addition, the sources of the two POX enzymes are not the same, and the reported kinetics have substantial differences. For example, the *K*_m_ for oxygen with POX from *P. chrysosporium* is 1.2 mM [30], while that for *T. multicolor* is 0.09 mM [66], and therefore, we might expect the system with *P. chrysosporium* to be more responsive to oxygen concentration over the dynamic range achieved with an oxygen headspace but at perhaps a lower catalytic efficiency and in the presence of an additional diffusion barrier, i.e., the bacterial cell wall.

The time course of the 200-ml reactions was also followed by HPLC and showed good correlation with the NMR study (Fig. 6). Analyses showed a composition of 40 ± 3% glucose and 63 ± 6% glucosone at 4 h and complete conversion of glucose to glucosone at 10 h (106 ± 1% glucosone), and the glucosone remained constant with extended incubation (100 ± 10% glucosone at 25 h). The stoichiometric conversion indicates that the POX reaction in resting cells can be fully decoupled from glucose central metabolism. Similarly, the reaction of GDH from *Thermoplasma acidophilum* coupled to an alcohol dehydrogenase from *Lactobacillus kefir* resulted in 98% conversion of cinnamyl aldehyde with 1.05 equivalents of glucose as sacrificial co-substrate [41]. Reactions in this case were in phosphate buffer (300 mM, pH 7) using *E. coli* cells stored at − 20 °C prior to catalysis. These results are in contrast to reactions with resting cells in M9 medium, minus a nitrogen source, where inorganics in the medium are able to support central metabolism, thus providing for the regeneration of NADPH [39] in the presence of supra-stoichiometric amounts of glucose.

Recognizing that a rotational mode of mixing is not the only option, we performed preliminary experiments using 200-ml reactions but with the bioreactor only partially inflated with 100% oxygen so that it is stable on a flat surface and allows consistent mixing on a rocking-motion-type incubator without the need for a form-fitted support. This configuration also leads to complete conversion of 10% glucose in 18 h, and the mode of mixing is more closely related to the wave-induced mixing used in previous reports [42, 67–69]. Complete glucose conversion indicates that the pillow reactor method used here is robust for these overnight lab-scale preparative syntheses, probably due to the wide window of opportunity to harvest the reactions, as seen with the form-fitted supports where reactions are complete in 10 h and the product remains stable for at least 25 h (Fig. 6). Since all the carbon from glucose can be accounted for in the product glucosone, it is a reasonable conclusion that no respiration occurs during the reaction, meaning there cannot be any accumulation of CO_2_ in the reactor. Therefore, the reactor headspace composition and pressure remain constant for the duration of the reaction (for an initial headspace composition of 100% O_2_), as the pillow deflates with oxygen consumption, thus remaining at 1 atm partial pressure of O_2_. Accordingly, the reaction conditions are well defined and reproducible when additional parameters, such as temperature, rotational speed, and rotational orbital diameter are fixed.

The overriding theme of the approach used in this study is the convenience of using an inexpensive disposable pillow reactor that requires only a single charge of headspace, which is simply accomplished, for whole-cell catalysis. This accommodates experimental research design because multiple simultaneous reactions can be run unattended and without the encumberments of electrical attachments and gas plumbing (as with sparged stirred-tank reactors). For example, 4 reactors can be easily accommodated in a rotary incubator with a 30 × 18-in. platform. With 3 shakers available, we were able to simultaneously test 4 different reaction conditions with 3 replicates (see Figs. 3 and 4). The scale of the reactions also permits the convenient synthesis of hundreds of grams of product overnight (e.g., approximately 240 g of product from 12 reactors, each containing 200 ml of 10% glucose, Fig. 6). The application described here applies to relatively short reaction times (overnight) using a reaction medium deficient in both nitrogen and inorganics necessary for general metabolism. Microbial contamination is not observed nor is it expected under these non-growth conditions and short reaction times. This approach is similar in concept to that of Whitesides et al., who used bubbles of bubble wrap (typically used as packing material) as sterile containers for storage, performing analytical procedures, and growing microorganisms [43], and we suggest that similar adaptive technologies might be applied here at significantly increased volume and with the added benefit of a ready-made resealable port.

## Conclusions

To the best of our knowledge, this is the first report of using inflatable packaging pillows with resealable ports as convenient, inexpensive, and effective bioreactors. The reactors provide gentle high surface area mixing suitable for both systematic research studies and for laboratory-scale preparative syntheses using non-growing *E. coli* whole cells. Parallel reactions permitted study of the effects of headspace composition (i.e., air vs 100% oxygen), cell density, exogenous catalase, and reaction volume in the oxidation of 10% glucose. When there is insufficient catalase, reactions are incomplete and result in formation of D-arabino-1,4-lactone and formic acid. For the preparative synthesis of glucosone, only a single charge of oxygen is necessary for complete reaction overnight. The simplicity of the reactor allows flexibility in research experimental design (from single-reactor studies, to more complex studies requiring multiple reactors) and in the scale of preparative syntheses. Incubators commonly used, such as rotary shaking or rocking-motion-type, are suitable for mixing the contents of the pillow reactors, and therefore, the methods presented here could be easily implemented and adapted for diverse applications in other laboratories.

## Supplementary Information

ESM 1 **Additional file 1: Figure S1.** A sheet of Kydex® V thermoplastic is notched (right) before pressing into a mold of an inflated pillow made with plaster cloth gauze. The shape of the formed thermoplastic is fine-tuned using a heat gun and trimmed to fit a fiberglass container (left). **Figure S2.** NMR assignments of product from 200-ml scale reactions. The labeling is in accordance with labeling in Fig. 1 where the letter indicates the anomer of glucosone and the number subscript the carbon of that anomer. The lower panel shows results obtained with first-round cells, and the upper panel is with recycled cells. (PPTX 560 kb)

ESM 2 Additional file 2: Movie S1. Demonstration of the pillow reactor. (MOV 17111 kb)

## Abbreviations

POX: pyranose 2-oxidase
GMC: glucose-methanol-choline
CAZy: carbohydrate active enZymes
AA3: auxiliary active family 3
NMR: nuclear magnetic resonance
AL: D-arabino-1,4-lactone
AA: D-arabinonic acid
MFG: molded fiberglass
TB: terrific broth
α-Glu: α-D-glucose
β-Glu: β-D-glucose
