# Supplementary figures and images for: Repurposing Inflatable Packaging Pillows as Bioreactors: a Convenient Synthesis of Glucosone by Whole-Cell Catalysis Under Oxygen
Source: Appl Biochem Biotechnol. 2020 Nov 13;193(3):743–60. doi: 10.1007/s12010-020-03448-x (PMC7910265; doi:10.1007/s12010-020-03448-x)

## Slide 1
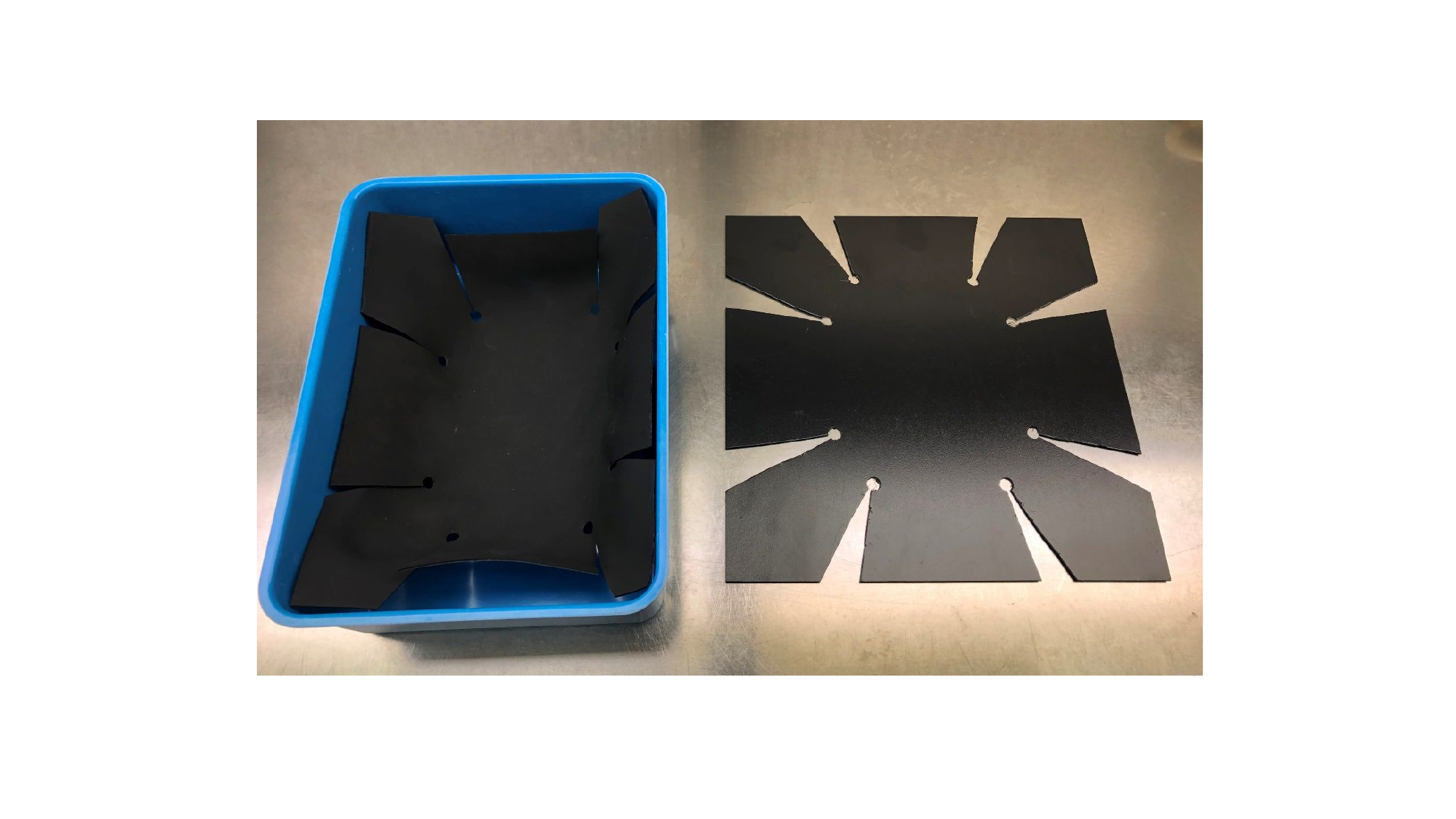

## Slide 2
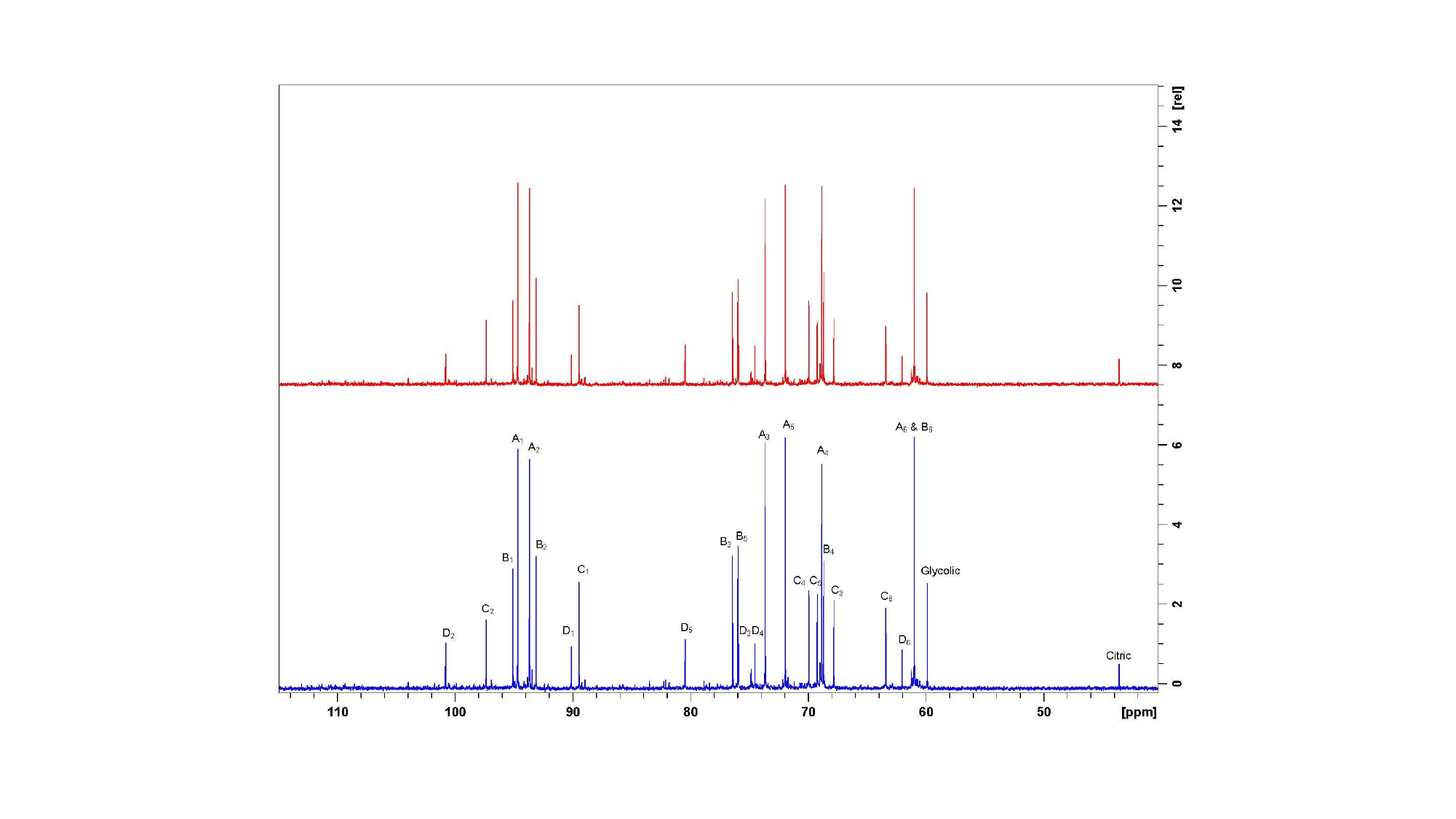

Supplement: Supplementary file 1 — Additional file 1: Figure S1. A sheet of Kydex® V thermoplastic is notched (right) before pressing into a mold of an inflated pillow made with plaster cloth gauze. The shape of the formed thermoplastic is fine-tuned using a heat gun and trimmed to fit a fiberglass container (left). Figure S2. NMR assignments of product from 200-ml scale reactions. The labeling is in accordance with labeling in Fig. 1 where the letter indicates the anomer of glucosone and the number subscript the carbon of that anomer. The lower panel shows results obtained with first-round cells, and the upper panel is with recycled cells. (PPTX 560 kb) [file 12010_2020_3448_MOESM1_ESM.pptx]
